# Improvement of the photoelectric dye sensitized solar cell performance using Fe/S–TiO_2_ nanoparticles as photoanode electrode

**DOI:** 10.1038/s41598-024-54895-z

**Published:** 2024-02-28

**Authors:** Chou-Yi Hsu, H. N. K. Al-Salman, Zaid H. Mahmoud, Rawaa Mahmoud Ahmed, Amir F. Dawood

**Affiliations:** 1 Department of Pharmacy, Chia Nan University of Pharmacy and Science, Tainan, Taiwan; 2 Pharmaceutical Chemistry Department, College of Pharmacy, University of Basrah, Basrah, Iraq; 3 Chemistry Department, College of Sciences, University of Diyala, Baquba, Iraq

**Keywords:** DSSC, XPS, Photolysis, Photoanode, Incorporating, Fe/S–TiO_2_, Chemistry, Nanoscience and technology

## Abstract

A sulfur nanoparticles-incorporated iron-doped titanium oxide (Fe/TiO_2_) with different ratio was successfully synthesized by photolysis method and utilized as effective photoanode in dye sensitized solar cell (DSSC) application with N719 dye. The photolysis method was contained the irradiation of the Fe, S and Ti mixture solution with 15 W source irradiation, and then calcined the formed precipitate. The DSSCs fabricated with Fe/S–TiO_2_ photoanode appeared an improved solar-to-electrical energy conversion efficiency of 6.46, which more than pure TiO_2_ (3.43) below full sunlight illumination (1.5 G). The impact of Fe content on the total efficiency was also inspected and the Fe content with 6% S–TiO_2_ was found 5 wt%. Due to the improved the efficiency of solar cell conversion of Fe/S–TiO_2_ nanocomposite, it should be deemed as a potential photoanode for DSSCs with high performance.

## Introduction

**Figure 1:**
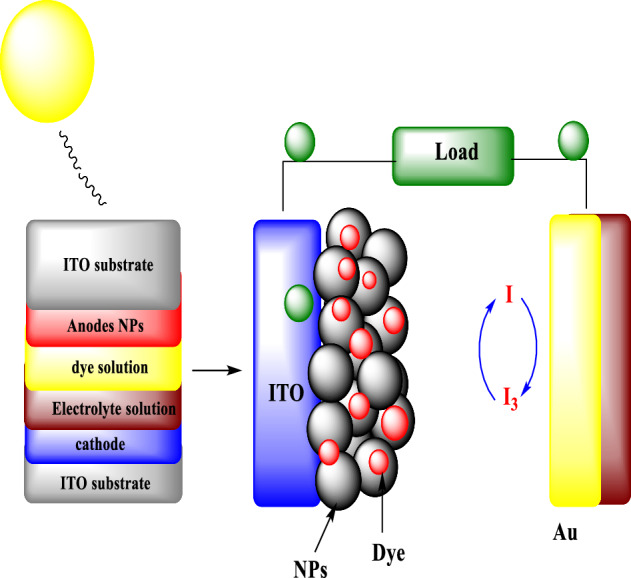
Schematic diagram DSSCs working.

O’Reagan and Gratzel primarily introduced dye sensitized solar cell (DSSC) in 1991^1^. Then, DSSC has been widely realized and deemed as most auspicious exchange of silicon solar cell^2^. DSSCs has been enticed authors due to that the low cost fabrication with high efficiency, eco-friendly fabrication process and excellent electrochemical and spectrum features^3,4^. The DSSC typical contain from mesoporous photoanode, counter electrode, dye and electrolyte solution. When DSSCs are lighted via solar light, the molecule of dye will oxidized via transporting the electrons from it to semiconductor that anchored on photoanode. After that, these materials capture electrons in its CB and then transfer it to counter electrode during outer circuit. At same time, electrolyte solution rejuvenate dye molecule via transporting electrons, which causes oxidation it^5–7^. Lastly, via accepting the electrons arriving during the outer circuit, the electrolyte that oxidized is again rejuvenate at counter electrode^8^. All these process are restore to support continuous product through the load^9^. Figure 1 appear the schematic of DSSC. Since the anode part adsorbs the dye molecule with transport of photo produced electrons that directly locate the efficiency of power conversion and DSSC photon current density. So, its necessary process to choose a suitable material for fabricating of photoanode^10^. Titanium dioxide is most consider utilized material for production of hydrogen, water and air purification, and photoanode fabrication because of many features such as low cost, nontoxicity, thermal stability, high efficiency of energy conversion^11–13^. A lone disadvantage in TiO_2_ is use a small part of solar light, approximately 10% in ultraviolet region due to its side band gap (3.2 eV), which cause low conversion of power^14^. However, the authors and researchers are giving their labors to design bandgap features of TiO_2_ via bimetal incorporating, which that DSSC efficiency can be improved via effective using of perfect solar spectrum^15^. In last studies an extended work on improved DSSC performance under irradiation of solar cell with (Zn, Ni, Fe, Cu) incorporated (C, N, F, S, B) incorporated, bimetals (Pd/Pt, Fe/Nb) incorporated and (Al/N, Cu/N, Ag/S) co-incorporated anatase TiO_2_ anode have been studied^16–20^. Until now, a maximum conversion efficiency was recorded by KaKiage et al.^21^ and achieved to 14.3% using TiO_2_ incorporated by mesoporous metal. Metal ions modulation into matrix of TiO_2_ could change the band gap and move its edge of optical absorption to higher wavelength, lead to boost photocurrent and inhibition the electron–hole recombination in DSSC. In ions incorporated TiO_2_ band gap, red shift is occurred because of electrons transferring from metal ions to CB of TiO_2_^22^. However, metal ions incorporated TiO_2_ has some hitches such as thermal instability and low IPCE, also change in TiO_2_ band gap was not eminent^23^. Moreover, the incorporating TiO_2_ by bimetal ion pairs could support lower band gap, thermal stability, improved surface area and high adsorption of dye. Accordingly, the delay recombination process of charge carrier, improved Jsc and then PCE^24^. Lately, good efficiency with 11.7% PCE was obtained for Cu/N incorporated TiO_2_ based DSSC^25^. Enhanced PCE with S incorporated TiO_2_ NFs based DSSC was recorded by Mahmoud et al.^26^. Zolfaghari et al.^27^ have synthesized Fe/S–TiO_2_ for degradation methylene blue dye. Hamadanian et al.^28^ have synthesized F/S co-incorporated TiO_2_ NPs by sol–gel method for photodegradation application of dye in aqueous solution. Photolysis has been consented as one of the multilateral mechanism for TiO_2_ synthesis, as it permit to give some of coveted structural parameters such as uniform morphology, low particle distribution and good porosity^29^. From previously reported via authors, this is carry on that Fe/S co-incorporated TiO_2_ could improve DSSC performance via improving properties like light trapping, dye adsorption and light absorption. In this study, we offer cheap and effective method for prepare wt%Fe/0.1 wt% S co-doped TiO_2_ NPs using photolysis method and their successful doping in DSSCs application. To the best of author’s recognition, Fe/S co-incorporated TiO_2_ has not been investigated using photolysis method yet, as a anode for DSSC application. Impact of iron and sulfur on the activity of anatase was studied and characterized.

## Experimental

### Materials

All reagents and materials were analytical grade and used without future purification. Ferric nitrate nonahydrate [Fe(NO_3_)_3_⋅9H_2_O], thiourea [CH_4_N_2_S] were supplied from Sigma Aldrich, while titanium chloride (TiCl_4_), ethylene glycol (EG), potassium iodide (KI), iodine (I_2_) and ruthenium N719 dye were procured from Merck Co.

### ***Synthesis of wt% Fe/S–TiO***_***2***_*** nanoparticle***

**Figure 2:**
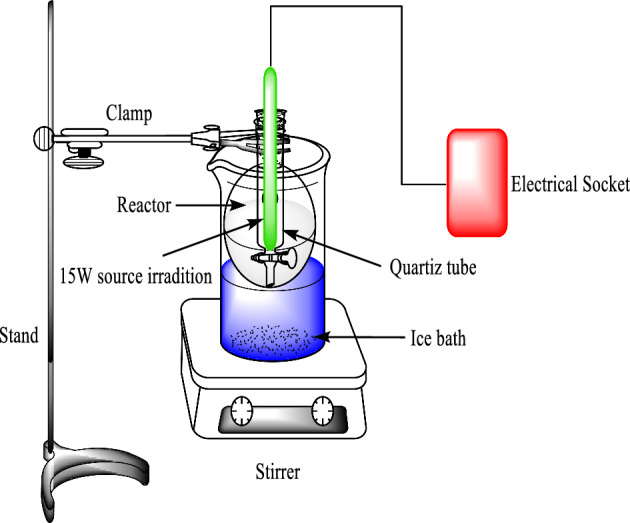
Manual irradiation system.

Photolysis method was depended for preparation of pure and incorporated TiO_2_ NPs. Typically, solution A was got via mixing 10 ml TiCl_4_ and 90 ml distilled water under continuous magnetic stirring at 15 °C. After that, solution B was prepared via mixing necessary quantity of thiourea (0.1 wt% relative to TiO_2_) and Fe(NO_3_)_3_⋅9H_2_O (1, 3 and 5 wt% with respect to TiO_2_). Then, the solution A was added to B under continuous magnetic stirring at 15 °C. individually, 200 ml of mixture was transferred to reactor of manual irradiation system (Fig. 2). The full procedure was reported by Zaid et al.^30^. The mixture was irradiated for 90 min under dropped 5ml of EG. The light brown precipitate was isolated by decantation method and washed several times via ethanol, acetone and distilled water. Finally, it dried and burned at 550 °C for 2 h.

### DSSCs fabrication

Firstly, the pure and Fe/S–TiO_2_ pastes were prepared via blending suitable quantity of EG and absolute ethanol with dry nanopowders independently^31^. To obtain homogeneous slurry of coveted viscosity, the prepared pastes were crushed continuously before using it. After that, ITO conductive glass with dimension (2*3) cm^3^ were taken and cleaned with ethanol by ultrasonic device for 30 min. Then, the cleaned ITO was dipped in TiCl_4_ solution for 30 min to made blocking layer. The DSSC active area were determined using scotch tape. The TiO_2_ and Fe/S–TiO_2_ pastes were precipitated via used doctor blade method^32^. The fabricated photoanodes were dried at 50 °C for 30 min and then, it calcined for 3 h at 500 °C. Via following the same procedure as mentioned above, the prepared photoanodes were again handled with titanium chloride to increase surface area, dye adsorption and reduce recombination rate, then calcined at 500 °C for 45 min. After that, all the fabricated photoanodes were left to cool at room temperature, before dipped in N719 dye solution (0.5 mM) for 24 h. The thickness of photoanodes were measured using profilometer SJ-210 and it was 20 μm. Coating prepared Au NPs on the ITO substrate surface produced counter electrode. Finally, the anode and cathode were sandwiched between 0.5 M iodine electrolyte solution that injected using micro-syrine in the void space. The fabricated solar cell were characterized by recording the current–voltage curve under light source with 100 mW/cm^2^.

## Results and discussion

### Structural characterization

**Figure 3:**
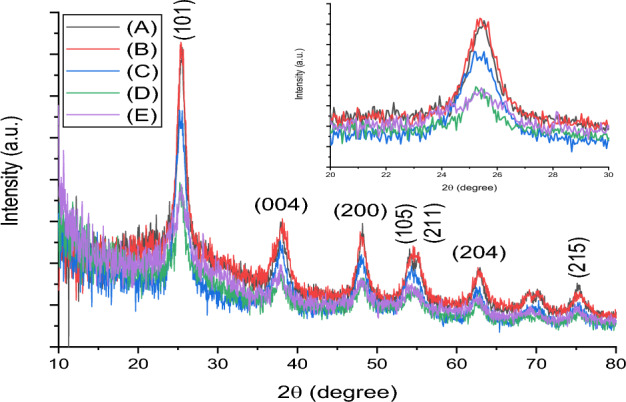
XRD of (**a**) TiO_2_, (**b**–**d**) 1,3 and 5 wt% Fe/S–TiO_2_.

The XRD patterns of pure and wt% Fe/S co-incorporated TiO_2_ NPs are shown in Fig. 3. All results have appeared anatase phase of TiO_2_, which corresponding to JCDPS no. 84-1286. However, the results no shown any diffraction pattern peak assign to Fe/S, which indicating the homogenous distribution inside the TiO_2_ crystal structure. In addition to, the XRD results of pure TiO_2_ has appeared another phase back to rutile at 35.6° diffraction peak. From XRD results, it clear appear that was not any considerable perversion in all diffraction peak position proposing effective addition incorporating of Fe/S in TiO_2_ crystal lattice^33^, whereas the DSSCs performance will depend on the Fe dopant concentration because using fix S concentration. Furthermore, the scattering impact is decrease with reduce Fe concentration leading to reduce in plasmonic impact. On the other hand, the increasing Fe content lead to increase agglomeration and this causing boost of particle size, reduce active area for adsorption N719 dye, and hence low DSSC efficiency. The crystallite size of prepared materials were investigated using Scherrer equation^34^ depending on prominent diffraction peak assign to (101) peak, and the results are summarized in Table 1. Among all prepared materials, the results shown that the 3 wt% Fe/S co-incorporated TiO_2_ has least crystallite size, which proposes highest surface area. The results shown that the increasing the content of Fe/S lead to decrease in crystallite size, which may be back to segregation of dopant on the TiO_2_ bonders, causing particles become hampered^33^.

**Table 1:** Size particles of pure and wt% Fe/S–TiO_2_.

Sample	2θ of crystal plane (101)	FWHM	D (scherrer) (nm)	Energy gap (eV)	Surface area (m^2^/g)
TiO_2_	25.46	0.670	13	3.24	78
1 wt% Fe/S	25.39	0.963	9	3.04	116
3 wt% Fe/S	25.25	1.593	5	2.91	126
5 wt% Fe/S	25.31	1.210	7	3.14	55

**Figure 4:**
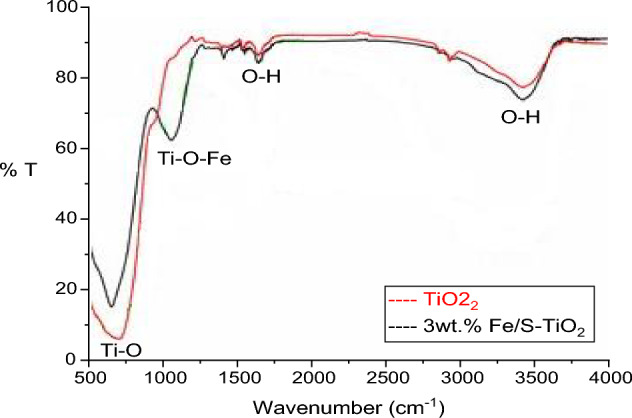
FTIR of pure and 3 wt% Fe/S–TiO_2_.

Figure 4 is displayed the FTIR spectrum of pure and 3 wt% Fe/S co-incorporated TiO_2_ NPs. Two peaks located at 1640 and 3410 cm^−1^ assign to bending and stretching vibration modes of OH for absorbed H_2_O molecules^35^. The results appeared that the OH peaks intensity be more in Fe/S co-incorporated, which mention to boost content of absorbed water. moreover, pure TiO_2_ spectrum shown a broad band centered at 1075 cm^-1^ was because of Ti–O–Ti stretching vibration mode, which indicates TiO_2_ formation^36^. Furthermore, the results illustrated a slight boost and shift in peak intensity for Fe/S doped case compared pure TiO_2_. In addition to, there are not any peaks back to Fe/S may be back to that the fraction of Fe/S was little, as well as, the atomic mass of S is less than Ti^37^. The FTIR spectrum confirm clearly the inter of Fe/S atoms in the structure of TiO_2_, and the results are in agreement with XRD.

**Figure 5:**
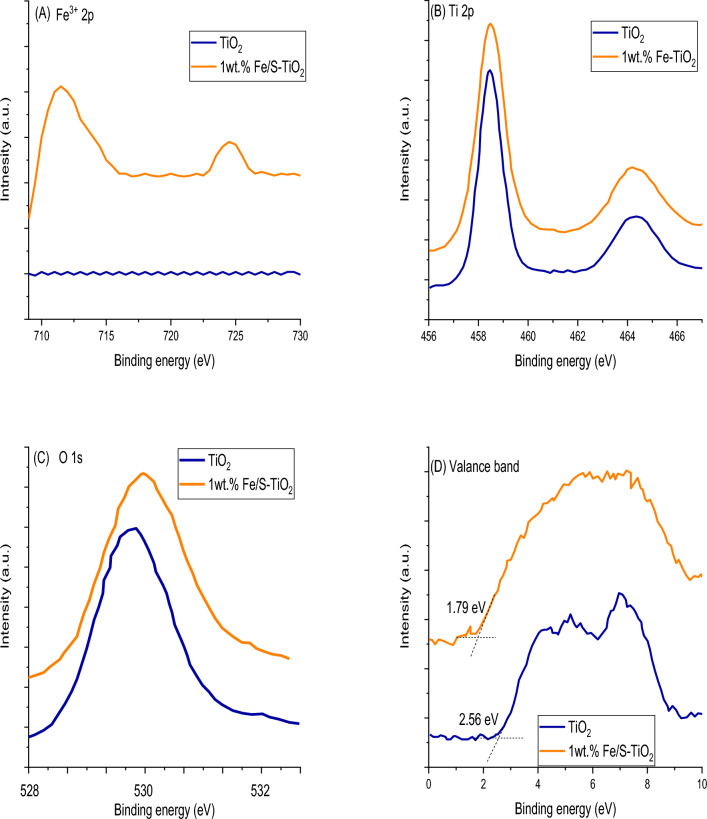
XPS of (**A**) Fe 2p, (**B**) Ti 2p, (**C**) O 1s and (**D**) valance band spectra of pure and 1 wt% Fe/N–TiO_2_.

**Figure 6:**
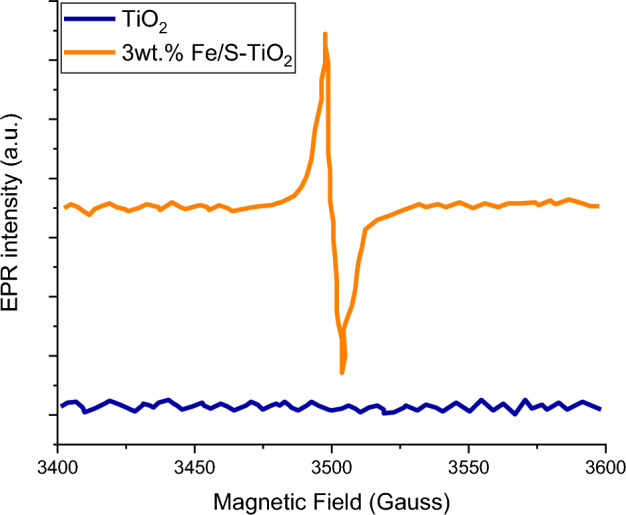
EPR of pure and 3 wt% Fe/S–TiO_2_.

The electronic state and chemical structure of pure and 1 wt% Fe/S co-incorporated TiO_2_ are investigated by XPS and shown in Fig. 5a–d. The spectra of XPS can discover the chemical binding of surface for 1 wt% Fe/S-TiO_2_ nanostrucutre. The spectra was enrollee utilizing Al K_α_ radiation with energy of 26 eV, as well as, it was calibrated via C 1s peak as a referencing at 284.6 eV. As demonstrated in Fig. 5a, the predominant XPS of Fe 2p peaks for Fe/S-TiO_2_ centered at 725.1 and 711.5 eV, which are corresponding to Fe^3+^ 2p_1/2_ and 2p_3/2_ binding energies, respectively^38,39^, and this detected that Fe^3+^ is the dominant charge state of iron state in Fe/S–TiO_2_. Comparing with pure TiO_2_ nanoparticles, Ti 2p spectra of Fe/S–TiO_2_ appeared no considerable difference (Fig. 5b), which mentioned there is not trivalance state for Ti that existence on the surface. However, this technique cannot reveal if there is and tri or tetra valance of Ti in the prepared compounds, for this we used the ERS to investigate the + 3 in it. The spectra of EPR were recorded at 25 °C using Bruker ER 200D-SRC electron spin resonance. This technique supplied elaborated information for the species and nature and their connection symmetries in the solid state. Always, the Fe^3+^ appear a broad indication about g = 1.94 due to it substituting the Ti^4+^ ions in crystal lattice of TiO_2_^39^. The EPR spectra of Fe/S-TiO_2_ expose a broad signal about g = 1.94, which illustrate the incorporating of Fe^3+^. Depending on the literature and reports, the Ti^3+^ will appear an acute EPR signal in TiO_2_^40^. The EPR spectra of synthesized TiO2 and Fe/S-TiO_2_ are shown in Fig. 6, and both show a narrow signal at g 1.94, which mentioned the existence of trivalance state of Ti in TiO_2_. These valance state were initiated because of the reduction role of UV radiation during synthesis of TiO_2_ and Fe/S–TiO_2_. briefly, the Fe/S–TiO_2_ compound, not only appear the broad EPR indication back to Fe^3+^ incorporating, but also shown a narrow peak back to the existence of Ti^3+^, and these results sure the co-incorporating of Fe^3+^ and Ti^3+^ in Fe/S–TiO_2_. As shown in Fig. 5c, two peaks located at 532.1 and 529.9 eV assign to Ti–OH surface and Ti–O lattice groups respectively. the results shown that the Ti–O of Fe/S–TiO_2_ shifted 0.2 eV compared with pure TiO_2_ because of the interaction between Fe^3+^ and Ti^4+^ and the bonding of Ti–O–Fe. In addition to, the results appeared that valance band (VB) of TiO_2_ is shifted 1.0 eV after incorporating by Fe/S as shown in Fig. 5d. These results capable us to discuss and understand the DSSC mechanism performance by using Fe/S–TiO_2_ compared with TiO_2_, whereas the incorporating sample dodge any considerable recombination of charges and expedite the separation of e–h. Incorporating the TiO_2_ nanoparticles with Fe/S lead to blue shift of the VB edge and prompt a notice bandgap narrowing, as well as, the synergistic impact between Ti^3+^ and Fe^3+^ play main role in narrowing the bandgap and hence enhance the DSSC performance^41^.

**Figure 7:**
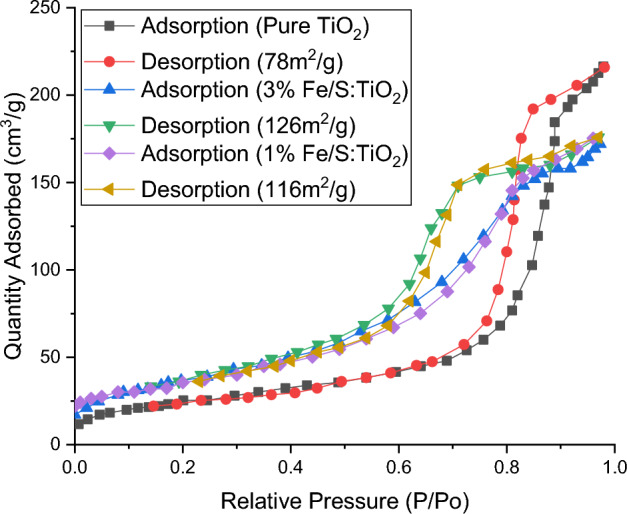
BET of pure and wt% Fe/S–TiO_2_.

The impact of insert Fe/S on the TiO_2_ surface area was investigated utilizing BET analysis and the results are shown in Fig. 7. The results demonstrated that all surface area of prepared compounds in the range of 55–126 m^2^/g and it tabled in Table. 1. As shown, the surface area are enhance with increasing Fe/S content until Fe/S doping to a specific limit because of heterogeneity formation and micropores opening. On the opposite side, the surface area of TiO_2_ are reduce with 5 wt% Fe/S dopant because of micropores closing and agglomeration of particles.

### Morphology characterization

**Figure 8:**
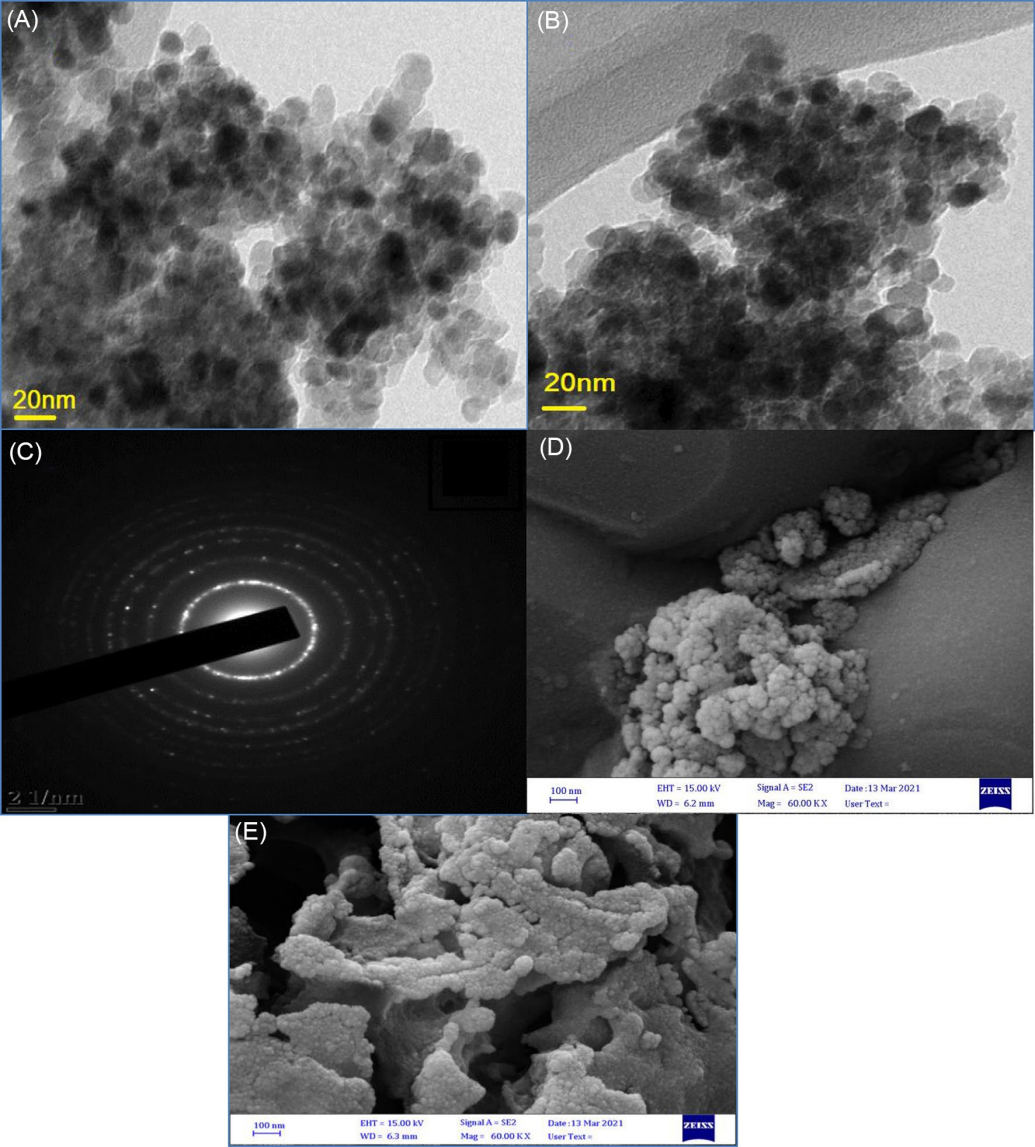
TEM of (**A**) pure TiO_2_, (**B**) 3 wt% FE/S–TiO_2_, (**C**) SAED pattern and FESEM (**D**) pure TiO_2_ and (**E**) 3 wt% Fe/S–TiO_2_.

The morphology of pure and wt.% Fe/S co-doped TiO_2_ were investigated by using TEM and FESEM. Figure 8a,b is shown the TEM images of pure and 3 wt% Fe/S co-incorporated TiO_2_ nanoparticles respectively. The results appear irregular shapes with many agglomeration, which lead to the particles more smaller. Moreover, the particle size of all prepared were construed utilizing Image J program. The size of particles for all prepared materials were predestine in the range of 10–35 nm. In addition to, the images shown reduce particle size with increasing Fe/S content, which indicate that the addition of Fe/S hinder the particle growth of TiO_2_ and hence, enhancing the surface area of prepared materials and thereby increasing the ability of dye loading. The selected area electron diffraction pattern of 3 wt% Fe/S–TiO_2_ is shown in Fig. 8c. The SAED image illustrate shining concentric rings assign to anatase diffraction plane. The FESEM images of pure and 3 wt% Fe/S–TiO_2_ nanoparticles are displayed in Fig. 8d,e respectively. The image show that the dopant sample have uniform distribution and has smaller size than pure TiO_2_. Moreover, the small particles and agglomeration of particle lead to boost the surface area of dopant samples till 3wt.% and hence it could improve the adsorption of dye, which causes higher Jsc of DSSC. However, more incorporating Fe/S content could led to reduce the surface area, lower dye loading, boosted recombination rate of charges and thereby low current density as appeared in 5 wt% Fe/N–TiO_2_.

### Optical properties

**Figure 9:**
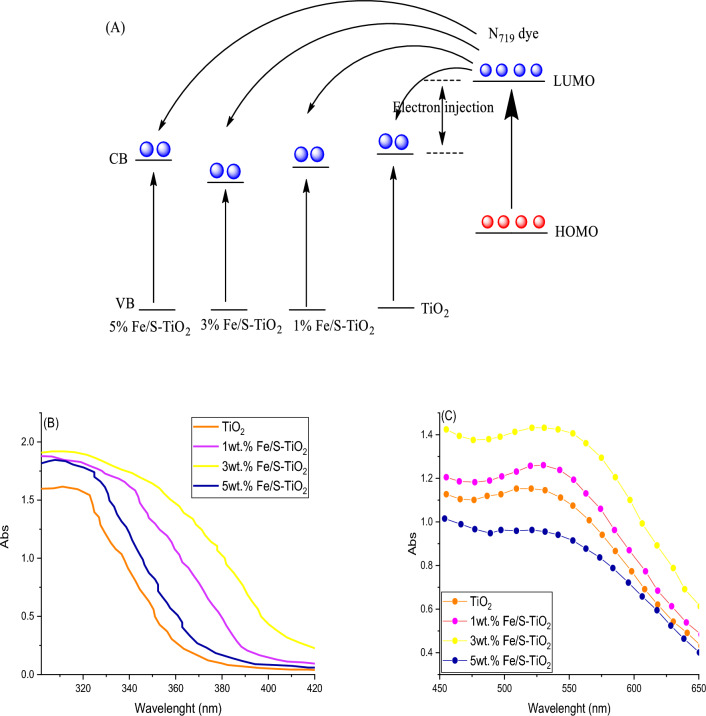
(**A**) Energy diagram schematic and electron injection process (**B**) Absorbance spectra, (**C**) absorbance spectra of N719 dye on pure and pure and wt% Fe/S–TiO_2_.

The optical properties of pure and wt% Fe/S–TiO_2_ were recorded by using UV–visible spectrophotometer (UV–Vis) and photoluminescence spectroscopy (PL). The injection of charge must be fast between photo-anode and N719 dye to evade recombination state. The appropriate edge level of band is requisite for decreasing recombination state. Moreover, the edge band of prepared photo-anodes must corresponding to the band gap of used dye for effective electron injection. Due to the N719 dye anchor with Ti atoms, the substitute Ti ions by Fe and S cations also effect on the adsorption of N719 dye because of different strengths binding between the Fe/S dopant and N719 dye. The incorporating Fe and S ions within TiO_2_ lattice structure alter the absorption edge position or made red shifts and decrease the energy gap and absorb a considerable part of visible light. It was located that utilized dopant employed to boost performance of device. The energy gap of Fe/S–TiO_2_ with among incorporating percentage utilizing using N719 was studied. It noted that incorporating altering the conduction band of TiO_2_. The red shift of TiO_2_ CB results in boosted injection electrons force, hence enhanced the efficiency of electron injection from dye LUMO to TiO_2_ CB. So, the transport of electron occur fast because of LUMO high potential level of N719 dye than TiO_2_ CB. thereby, results into the enhanced efficiency of charge collection and density of photocurrent (Fig. 9a). Incorporating attained via substituting Fe and S cations with Ti in crystal structure of TiO_2_ that has considerable impact on the trap states of oxide. The optical response of synthesized materials were investigated by UV–Vis spectra and the results are shown in Fig. 9b,c. The UV–Vis spectra are displayed in Fig. 9b. The results appeared that with increasing Fe/S content, the edge of peak absorption was transferred to higher wavelength (red shift) and the absorption boost to an optimum frontier because of the resonance of localized surface Plasmon^42^. On the opposite side, the red shift reduces with increasing Fe/S content (5wt.%), causes in reduced absorption and Jsc^43^. The band energies of pure and wt.% Fe/S–TiO_2_ were obtained from UV–Vis spectrum and calculated utilizing Kubelka–MunK function, and it summarized in Table 1. The results appeared that with increasing Fe/S content, the bandgap reduced because of the synergistic impact of Fe-3d hybriding and S species with Ti 3d, hence forming defect levels of localized over TiO_2_ VB. The mixed impact of hybriding led to reduce band gap and enhancement the red shift to high wavelength, hence improving their restraint effectively to the visible light^44^. Furthermore, the UV–Vis was utilized to determine the dye adsorption quantity on pure and wt% Fe/S–TiO_2_ photoanodes. Figure 9c demonstrates the pure and Fe/S–TiO_2_ adsorption spectra. The N719 dye adsorbed quantity was investigated utilizing the desorbed the N719 from the different pure and Fe/S doped TiO_2_ photoanodes with 0.001M solution of NaOH^45^. The results obtained that the 3 wt% Fe/S–TiO_2_ illustrates a special raised N719 loading, which may be back to high surface area and suitable particle size. On the opposite side, the loading of N719 reduced extremely with 5 wt% Fe/S–TiO_2_ because of boosted agglomeration and decreased surface area.

**Figure 10:**
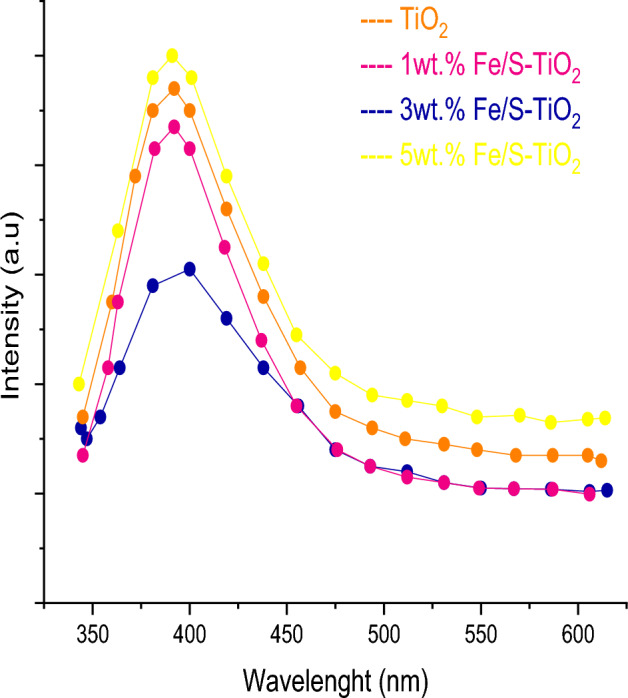
PL spectra of pure and wt% Fe/S–TiO_2_.

The PL analysis was used to study and investigate the recombination electron–hole state in synthesized nanocomposite and the results are shown in Fig. 10. The intensity of PL were decreased with increasing the Fe/S till 3 wt% dopant, appearing delay in recombination rate because of effective charges transport as the content ratio boosted, hence, increasing the electron injection at photoanode/N_719_ dye^46^.

### DSSCs performance

**Figure 11:**
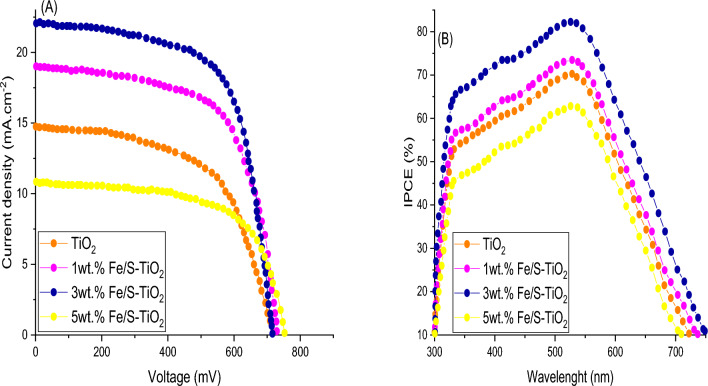
(**A**) J-V characteristic and (**B**) IPCE of pure and wt% Fe/S–TiO_2_.

The efficiency of power conversion and the execution of fabricated pure and Fe/S incorporated TiO_2_ photoanodes based DSSCs were investigated at standard condition (100 mW/cm^2^) via plotting current density as a function to applied voltage. The J-V characteristics which contain Voc, Jsc, Jmax, Vmax, FF and η of fabricated photoanodes were calculated using Eq. (1) and are summarized in Fig. 11a and Table 2. The results appeared that the highest PCE of 82.24% was obtained for 3 wt% Fe/S-TiO_2_ photoanode which was 70% higher than pure TiO_2_ photoanode based DSSC. The 3 wt% Fe/S doped TiO_2_ NPs has appeared appropriate particle size and proper bandgap energy lead to considerable improvement absorption of visible light and adsorption N719 dye and thereby demonstrated most enhanced Jsc. However, poor circuit density and low efficiency was appeared for 5 wt% Fe/S–TiO_2_ based DSSC because of boosted particle size and low adhesion of N719 dye. Figure 11b shows the conversion efficiency of incident photon to current spectra for different fabricated DSSCs that measured as a function of wavelength. Generally, the spectra of IPCE supply a functional acquaintance around the photo response from fabricated DSSC for a fixed incident light wavelength^47^. The results appeared that the photogenerated current arrives to the maximum peak value in blue-green zone from the visible light spectrum, which assign to N719 dye distinctive absorption peak. The current density Jsc values were also estimated form IPCE spectrum by utilizing the following equation:

$${J}_{sc}=\int qF(\lambda )IPCE(\lambda ) \partial \lambda$$

where, q is the charge of electron, while the F(λ) is represented to flux of incident solar. By utilizing the mention relation, the Jsc value was calculated and its found 16.67 mA/cm^2^, which was less than the Jsc (19.53 mA/cm^2^) that estimated from J–V plot, which may be assign to two facts: (1) the measurements of IPCE were commonly carried out under light with low intensity, hence the generation and separation of charges with low efficient, (2) high reflectivity of Au electrode supplied extra light gathering of the Fe/S–TiO_2_, commanding to improvement Jsc and PCE. The results appeared that 3 wt% Fe/S–TiO_2_ NPs showed a top improvement in IPCE about 82% compared with other fabricated DSSCs, which assign to the smaller particle size, increasing surface area, higher adsorption of N719 dye and enhance Jsc.

**Table 2:** photovoltic performance parameters of DSSCs based pure and wt.% Fe/S-TiO_2_.

Sample	J_sc_ (mA/cm^2^)	V_oc_ (mV)	FF	η	IPCE (%)
TiO_2_	12.50	457.45	0.6	3.43	70.48
1 wt% Fe/S	16.97	475.33	0.65	5.24	73.56
3 wt% Fe/S	19.53	501.29	0.66	6.46	82.75
5 wt% Fe/S	9.44	475.23	0.64	2.87	63.11

### Interfacial charge transfer mechanism

**Figure 12:**
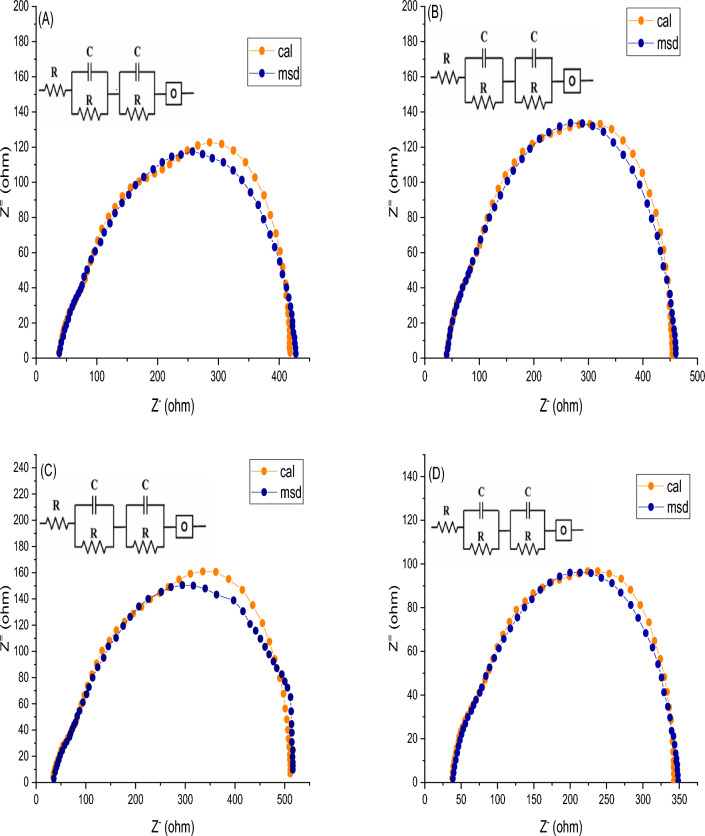
Impedance spectra of (**A**) pure TiO_2_, (**B**) 1 wt% Fe/S–TiO_2_, (**C**) 3 wt% Fe/S–TiO_2_ and (**D**) 5 wt% Fe/S–TiO_2_.

**Figure 13:**
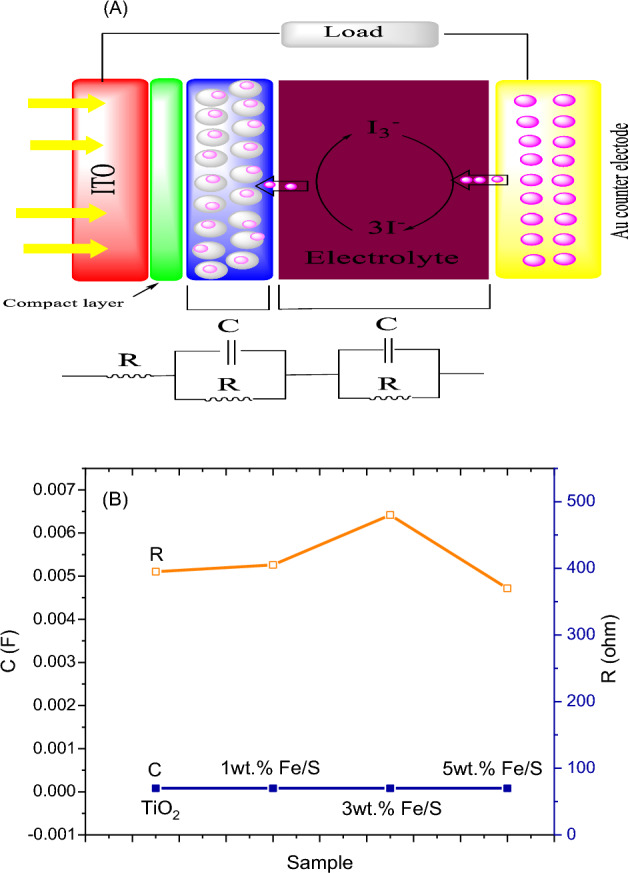
(**A**) Schematic of transmission line mode and (**B**) Variation in charge transfer capacitance and polarization capacitance with Fe/S content.

DSSC J–V mensuration is the basic measurement that give us on the fabricate device overall performance in term of it efficiency, but it give us little acquaintance around the DSSCs individual resistance and bordering factors which impact on the DSSC performance. The electrochemical impedance spectral (EIS) was employed to investigate the transfer of interfacial charge that present in fabricated of pure and wt% Fe/S doped TiO_2_ internal equivalent circuit contain contact resistance in frequency of 1 MHz–1 Hz. The plots of Nyquist of pure and wt% Fe/S co-doped TiO_2_ are appeared in Fig. 12. Generally, the plots contain three semicircles related to the different transport process of charges. Its consist from the first semicircle in high, low and intermediate frequency regions assign to charge transfer at Au electrode, electro-active species diffusion in electrolyte and transport of electron in prepared photoanode and the reverse reaction at interface of anode/electrolyte respectively. ZSimpwin software was used to simulated the DSSC impedance data and plotted employing model of equivalent circuit as appeared in Fig. 12a–d. The plot shown (Fig. 13a) that model of circuit congruous DSSCs phenomena. The recombination process is link to the mid frequency impendence that participate more in the impedance. So, the rate of recombination is smaller, when the resistance of mid frequency polarization is large and vice-versa. The results shown (Fig. 13b) that, the resistance of overall polarization boost with increasing Fe/S content. The Fe and S cations resort to substitute the atoms of Ti in crystal lattice instead of take place in site of interstitial because of atomic radius convergence between Ti and Fe^48,49^. The existence of Fe/S ions appeared a positive shift of VFB in plot of MotteSchottky, which causes in decrease recombination and increasing transfer of electrons density^50^ and trapped state and then reduce mid frequency impedance^51^. So, the optimum content is 3 wt% Fe/S that supply balance between mid-frequency and ratio of injection to increase DSSC performance.

## Conclusion

A slick method to synthesis Fe/S–TiO_2_ by photolysis method to fabricate a good photoanode for DSSC performance was shown. The Fe/S-TiO_2_ nanocomposite was characterized by XRD, XPS, EPR, FTIR, BET, TEM, FESEM and EIS. The DSSC fabricated appeared an improvement solar-to-energy conversion efficiency with 9% compared with DSSCs assembled by using TiO_2_ as a photoanode below simulated solar irradiation of 100 mW/cm^2^. The improvement in the performance of photovoltic was fundamentally back to the Fe/S co-doped nanoparticles, which improved the absorption of visible light as a result of their light gathering characteristic because of the surface Plasmon impact. Moreover, it also mainly consolidate transport of interfacial charge, which delay the process of charge recombination. The better Fe/S content incorporated TiO_2_ to appear an effective photoanode was found to be 5wt.%. As well as, the Fe/S dopants helped to decrease the bandgap and shift the absorbance to visible region and also restrain the recombination of charge. All reasons that contain, Plasmon impact, reduce band gap and effective charge transfer to increase the DSSC conversion efficiency.

## Data Availability

All data generated or analyzed during this study are included this published article.
